# Ionic liquid gating control of RKKY interaction in FeCoB/Ru/FeCoB and (Pt/Co)_2_/Ru/(Co/Pt)_2_ multilayers

**DOI:** 10.1038/s41467-018-03356-z

**Published:** 2018-03-07

**Authors:** Qu Yang, Lei Wang, Ziyao Zhou, Liqian Wang, Yijun Zhang, Shishun Zhao, Guohua Dong, Yuxin Cheng, Tai Min, Zhongqiang Hu, Wei Chen, Ke Xia, Ming Liu

**Affiliations:** 1 Electronic Materials Research Laboratory, Key Laboratory of the Ministry of Education & International Center for Dielectric Research, Xi’an Jiaotong University, 710049 Xi’an, China; 2 Center for Spintronics and Quantum System, State Key Laboratory for Mechanical Behavior of Materials, School of Materials Science and Engineering, Xi’an Jiaotong University, Shaanxi 710049 Xi’an, China; 3 Materials Science Division, Argonne National Laboratory, 9700 Cass Avenue, Lemont, IL 60439 USA; 4 Institute for Molecular Engineering, The University of Chicago, 5640 South Ellis Avenue, Chicago, IL 60637 USA; 5 The Center for Advanced Quantum Studies and Department of Physics, Beijing Normal University, 100875 Beijing, China; 6 Synergetic Innovation Center for Quantum Effects and Applications (SICQEA), Hunan Normal University, 410081 Changsha, China

## Abstract

To overcome the fundamental challenge of the weak natural response of antiferromagnetic materials under a magnetic field, voltage manipulation of antiferromagnetic interaction is developed to realize ultrafast, high-density, and power efficient antiferromagnetic spintronics. Here, we report a low voltage modulation of Ruderman–Kittel–Kasuya–Yosida (RKKY) interaction via ionic liquid gating in synthetic antiferromagnetic multilayers of FeCoB/Ru/FeCoB and (Pt/Co)_2_/Ru/(Co/Pt)_2_. At room temperature, the distinct voltage control of transition between antiferromagnetic and ferromagnetic ordering is realized and up to 80% of perpendicular magnetic moments manage to switch with a small-applied voltage bias of 2.5 V. We related this ionic liquid gating-induced RKKY interaction modification to the disturbance of itinerant electrons inside synthetic antiferromagnetic heterostructure and the corresponding change of its Fermi level. Voltage tuning of RKKY interaction may enable the next generation of switchable spintronics between antiferromagnetic and ferromagnetic modes with both fundamental and practical perspectives.

## Abstract (web summary)

Energy efficient control of magnetization in antiferromagnetic materials is one of the key ingredients for antiferromagnetic spintronics. Here the authors demonstrate voltage control of RKKY interaction and magnetization switching in synthetic antiferromagnetic multilayers by ionic liquid gating.

## Introduction

Antiferromagnetic (AFM) spintronics, as a fast growing cutting edge field in spintronics, is of great importance for its tremendous potential in the next generation of spintronics applications^1–3^. Compared with traditional ferromagnetic (FM) spintronics, AFM spintronics has several advantages, such as high magnetic field (H-field) stability^2,4,5^, fast operation speed at THz^6–11^, and wide compatibility to metal, semiconductor, or insulators^2,4^. Contradictorily, the insensitivity of magnetic perturbation of AFM materials also limits the regulation of AFM spintronics by H-field^1,12^. Therefore, AFM spintronics is usually controlled by spin-polarized current^2,13,14^. However, this controlled way has a relatively large current density (10^6^ ~ 10^11^ A m^−2^), which causes heat generation, energy inefficiency, and speed limitations^4,13,15–18^.

In the recent decade, electric-field (E-field) regulation of antiferromagnetism has gained increasing attention for its high speed, compactness, and energy efficiency. To date, E-field control of exchange bias (EB) has been carried out^19–22^. Nevertheless, since the alternative spins at the AFM/FM interface are strongly pinned by AFM layer, these E-field control processes are usually confined at a low temperature^20,21^ or require an H-field assistance^22^. To overcome these difficulties, we considered the synthetic antiferromagnetic (SAF) multilayer as an easier way to manipulate AFM coupling^5,23,24^. In the SAF structure, two FM layers separated by a nonmagnetic (NM) metallic spacer layer have indirect interaction through interlayer exchange coupling (IEC)^24^. According to the Ruderman–Kittel–Kasuya–Yosida (RKKY) interaction theory^24–27^, whether the FM layers are ferromagnetically or antiferromagnetically coupled is determined by the thicknesses of NM layer^25^. Many researches regarding SAF heterostructures have been done^26–31^. However, none of them have focused on controlling RKKY interaction by E-field, which is actually very promising, considering its compactness and energy effectiveness.

Recently, the ionic liquid (IL) gating process for voltage modulation magnetism has drawn much attention. It can manipulate the interfacial magnetism of ultrathin metallic films by accumulating surface charge^32^; realize tri-phase transition by modulating oxygen vacancies in oxide thin films^33^; regulate magnetic properties by changing the electron density at the Fermi level^20,34^. This IL gating process has many superiorities over traditional multiferroics, such as room-temperature operation^32,35^, large magnetoelectric (ME) tunability^32^ and compatibility on various substrates^32,34,36^. What’s more, comparing with the high current density (~10^10^ A m^−2^) of spin transfer torque^37^ method or spin-orbit torque^18^ method, the circuit gating voltage (*V*_g_ < 5 V)^32,34,38^ for antiferromagnetism/magnetism manipulation can be satisfied more easily in regular electronic devices. Therefore, IL gating-controllable AFM spintronics is potential for better performance and extra manipulation degree of freedom^20^. Therefore, we expect the interfacial charge accumulation will influence the Fermi level of SAF heterostructure and tune RKKY interaction accordingly. What’s more, the voltage switching of AFM and FM modes may further bridge the large gap between the two important branches of spintronics—AFM and FM spintronics, indicating a tremendous application potential.

In this work, we choose 1, 3-diallylimidazolium bis (trifluoromethanesulfonyl) imide ([AAIM]^+^[TFSI]^−^, []^+^ used to mark the electrical property) as IL gating dielectric layer, which has abundant interfacial phenomena^20,32,35,39–41^. A series of FM/NM/FM SAFs with in-plane magnetic anisotropy (FeCoB/Ru/FeCoB) and perpendicular magnetic anisotropy (PMA, (Pt/Co)_2_/Ru/(Co/Pt)_2_) are fabricated, respectively. Vibrating sample measurements (VSM) reveal a clear Ru thickness (*t*_Ru_) dependence of RKKY interaction strength and demonstrates that the IL gating could introduce fascinating magnetic behaviors with a small *V*_g_ (<5 V). At room temperature, hysteresis loops transform electrically among single-loop, double-loop, and triple-loop patterns. In other words, parallel, antiparallel, and canted magnetic moment alignments of FM layers are switched by E-field as demand, corresponding to FM coupling mode, AFM coupling mode, and other canted modes in the SAFs. Besides, voltage control of dynamic domain switching is achieved in the perpendicular SAF through room-temperature Magnetic optical Kerr (MOKE) microscope, which is highly desirable in high-performance spintronic memory and logic devices^42,43^. Up to 80% of perpendicular magnetic moments manage to switch with a small-applied voltage of 2.5 V on (Pt/Co)_2_/Ru/(Co/Pt)_2_. In addition, the first principle calculation with changing the potential (Fermi energy) is taken to reveal the inner mechanism. We find that the IEC could be turned by the gating voltages from a negative value (AFM ordering) to positive (FM ordering) in some finite thickness of Ru, which turns out to be the reason of the intense control effect. Meanwhile, the changes of IEC strength under IL gating has been calculated in both qualitatively and quantitatively way for better understandings of magnetization transformations. This transforming technique between AFM and FM coupling modes is promising for voltage switchable AFM and FM spintronics.

## Results

### Microstructure and magnetic properties for SAF heterostructures

**Fig. 1:**
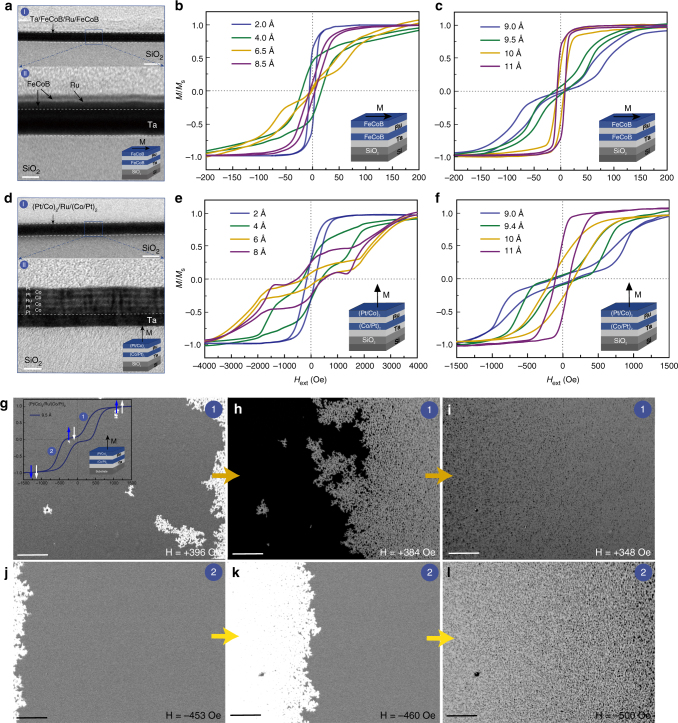
Microstructure and magnetic properties. **a** Cross-sectional TEM images for FeCoB (1.5 nm)/Ru (0.95 nm)/FeCoB (1.5 nm)/Ta (7.5 nm)/SiO_2_/Si structure. II are partial enlarged images for I. Scale bar in I, 20 nm. Scale bar in II, 5 nm. **b**, **c** are magnetic hysteresis loops for in-plane FeCoB/Ru/FeCoB SAF heterostructures with different Ru thickness. **d** Cross-sectional TEM images for (Pt 9 Å/Co 7.5 Å)_2_/Ru (0.95 nm)/(Co 7.5 Å/Pt 9 Å)_2_/Ta (3.5 nm)/SiO_2_/Si structure. II are partial enlarged images for I. Scale bar in I, 50 nm. Scale bar in II, 5 nm. **e**, **f** are Ru thickness dependence of magnetic hysteresis loops for out-of-plane (Pt/Co)_2_/Ru/(Co/Pt)_2_ SAF heterostructures. **g**–**l** Perpendicular dynamic magnetization reversal for (Pt 9 Å/Co 7.5 Å)_2_/Ru (0.95 nm)/(Co 7.5 Å/Pt 9 Å)_2_/Ta (3.5 nm) structure observed in polar MOKE mode. Scale bar, 50 μm. Figure (**g**–**i**) marked by 1 are the images when H-field decreases from +1000 to +0 Oe. Figure (**j**–**l**) marked by 2 are the images when H-field decreases from +0 to −1000 Oe

We tried to fabricate ultrathin films to guarantee an effective IL gating process^32^, but the test signals of samples were not impaired. As indicated by the microstructure and basic magnetic properties, our samples are free from the dead layer effect. Figure 1a displays the nanostructures of FeCoB trilayer layer (in-plane magnetic anisotropy) examined by a high-resolution transmission electron microscope (HR-TEM). The layered structure of FeCoB/Ru/FeCoB/Ta from top-to bottom can be clearly identified in the enlarged pictures (Fig. 1a II), which verifies clear and continuous interfaces among different layers. This is crucial for the RKKY interaction.

The magnetization state could be precisely controlled in these SAF heterostructures. Figure 1b, c show the Ru thicknesses (*t*_Ru_) dependence of FeCoB/Ru/FeCoB onto Si/SiO_2_ multilayers. It has been demonstrated both in experiment and in theory that RKKY interaction shows periodical oscillations in FM and AFM coupling when the thickness of NM layer changes^27,28,44^. In the in-plane system, when *t*_Ru_ changes from 2 to 6.5 Å, the indirect interaction between two FM layers gradually generates AFM coupling instead of FM coupling. In this case, easy-axis (EA) hysteresis loop shows a double S-shape and the main characteristic of the magnetic reversal exhibits a spin-flop transition behavior^44,45^. AFM strength, which is determined by the hybridization between the 3d bands of FM layers and the conduction band of NM layer^27^, is an oscillatory attenuation function as NM layer thickness increases^27,44,46^. When *t*_Ru_ increases to about 9 Å, the periodical oscillatory coupling comes to the second AFM coupling area, where we can obtain a weaker coupling strength that is easier to control with an applied voltage^27^. The different loop shapes of AFM-coupled samples (9 Å, 9.5 Å) are the results of a competition between AFM coupling, uniaxial in-plane anisotropy, and magnetocrystalline volume anisotropy^44^. If *t*_Ru_ keeps increasing to 10, 11 Å, hysteresis loop transforms to a single-loop that corresponds to a parallel or nearly-parallel state^28^.

From the application point of view, the trilayer structures of GMR and TMR accompanied with PMA have been attracting as much attention as memory cells in ultrahigh-density magnetic sensors and devices^28,47^. Therefore, we further studied the voltage control effect of SAFs with PMA based on (Pt/Co)_2_/Ru/(Co/Pt)_2_ structures. Figure 1d shows the TEM result of Co/Pt composites (with PMA). The layered structure of Pt/Co/Pt/Co/Ru/Co/Pt/Co/Pt/Ta from top-to-bottom is clearly identified in the enlarged pictures (Fig. 1d II); this is crucial for both RKKY interaction and PMA exhibition. Supplementary Fig. 1 shows the polycrystalline structure of Co/Pt system. Figure 1e, f display the *t*_Ru_ dependence of (Pt/Co)_2_/Ru/(Co/Pt)_2_ multilayers. The first AFM coupling interval is 0.4 to 1 nm, while the second oscillatory area for AFM coupling is around 2 nm (since films less than 1 nm are more effective for IL-gating-control, 2 nm data are not shown here). This perpendicular SAF configuration has PMA and IEC at the same time, making it different from a pure in-plane SAF structure. The PMA depends on Co 3d–Pt 5d interfacial hybridization^48,49^; the IEC between two FM layers ((Co/Pt)_2_ serves as a compound FM layer) depends upon the hybridization between the 3d bands (of Co layer) and the conduction band (of Ru spacer)^27^. The magnetic behavior of the present structure is an integration of both effects, so we let *t*_Ru_ be the only variation parameter to highlight the IEC.

AFM-coupled multilayers offer novel templates to study the magnetic behaviors. In the perpendicular SAF system, the 9.5 Å sample carries typical AFM coupling; based on it, we studied the dynamic magnetization reversal process as shown in Fig. 1g–l. Corresponding normalized hysteresis loop places in the top left corner of Fig. 1g. In this case, a relatively sharp spin-flop transition can be observed easily with MOKE microscopy. It is reported that if external H-field is strong enough to overturn the sublattice magnetization vectors in AFM systems, magnetic reorientation transition will happen in the easy-axis direction and specific transitional domain structures will form in the spin-flop region^45^. For the 9.5 Å out-of-plane SAF heterostructure, domain evolutions display two transition stages when H-field decreases from +1000 to −1000 Oe; this is the same as revealed by the hysteresis loop in Fig. 1g. There are two different kinds of spins in the SAF multilayer: surface spin (white arrow) and bulk spin (blue arrow)^50^. The surface spins on the outer side of FM layers (facing the surface or the buffer/substrate) are freer than the inside bulk spins, making themselves respond more easily to the external H-field^50^. Therefore, when H-field is around +380 Oe (surface spin-flop field, ${\mathrm{H}}_{{\mathrm{SF}}}^{\mathrm{S}}$), the active surface spins manage to rotate into a flopped state, generating the first phase of domain nucleation as well as the first phase of sharpening hysteresis loop. As H-field keeps decreasing and reaches the center of spin-flop field (${\mathrm{H}}_{{\mathrm{SF}}}^{\mathrm{B}}$, −460 Oe), the spin-flopped phase spreads throughout the whole system, leading to the second domain structure formation.

### IL-gating-control of RKKY interaction for in-plane and out-of-plane SAF heterostructures

**Fig. 2:**
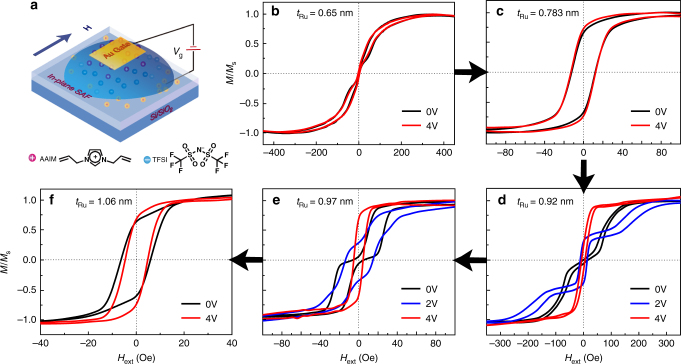
Controllable magnetic hysteresis loops switching by IL gating for in-plane FeCoB/Ru/FeCoB/SiO_2_/Si SAF heterostructures. **a** Schematic of IL gating magnetism modification process. **b**–**f** are in-situ VSM test of all samples while under IL gating modification. The black curves, blue curves, and red curves illustrate the ungated films, the films gated at 2 V, 4 V, respectively. The applying magnetic field was along easy axis (in-plane)

**Fig. 3:**
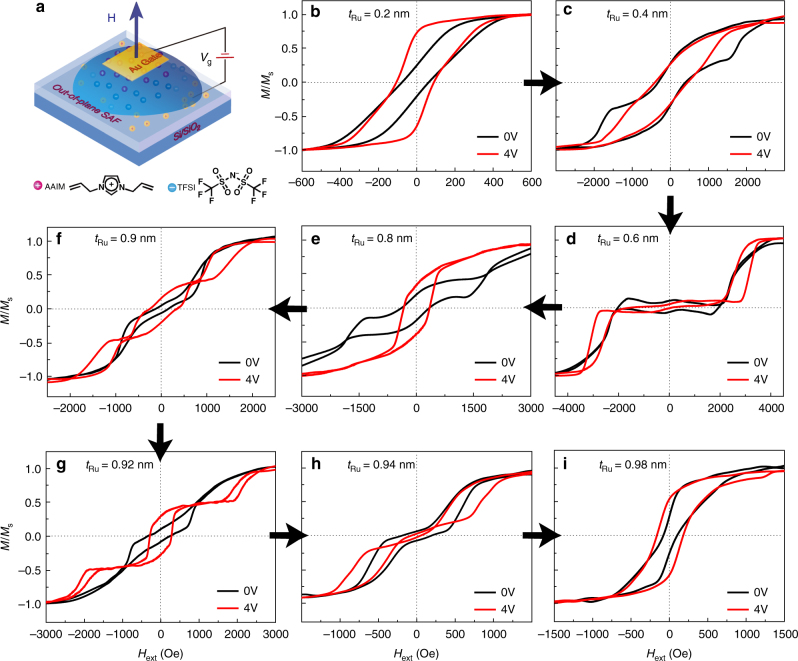
Voltage control of RKKY interaction for out-of-plane configuration with (Pt/Co)_2_/Ru/(Co/Pt)_2_ heterostructures. **a** Schematic of IL gating while the applying H-field was along easy axis (out-of plane). **b**–**i** are the corresponding in-situ VSM tests for different Ru thickness at 0 and 4 V, respectively. Both the RKKY interaction and IL gating modification in Co/Pt system are very sensitive to *t*_Ru_

IL gating of Au/[AAIM]^+^[TFSI]^−^/FM layer/Ru/FM layer/Ta/SiO_2_/Si heterostructures with both in-plane anisotropy and PMA were investigated by in-situ VSM measurements and the results are shown in Figs. 2 and 3. The IL gating configurations are shown in Figs. 2 and 3a, respectively. In the IL phase, the E-field driven anions and cations migrate toward the Au electrode and film electrode. Correspondingly, opposite charges accumulate at IL/film interface and form an electric double layer over a length scale about 3 nm, making the surface charge density *n*_s_ dramatically increases up to 10^15^ cm^−2^^32,41,51,52^. Accompanied by a strong interfacial E-field, this charge accumulation can lead to interfacial ME phenomena^32^.

This IL gating modification can be divided into two regions according to the critical voltage of current surge^32^. In our case, the electrostatic doping region is *V*_g_ < 1.1 V for FeCoB and *V*_g_ < 2.1 V for Co/Pt; the electrochemical reaction region is *V*_g_ ≧ 1.1 V for FeCoB and ≧2.1 V for Co/Pt, as calibrated in Supplementary Fig. 2. The electrostatic doping within the electrochemical window at the interface has a limited effect on the RKKY interaction, so we increase the applied *V*_g_ to electrochemical reaction region. During this process, ions of film plane are introduced to IL^32^, enabling a more effective regulation to the SAF structures. For simplification, only positive *V*_g_ is considered in this study. Basically speaking, the IL gating process should be carried out with inactive nitrogen gas protection^32^ or in vacuum condition^36^ to reduce the possibility of the chemical reaction. However, to the best of our knowledge, there has been none in-situ VSM measurements for IL gating process so far. Although we successfully develop in-situ VSM measurement here, it is still very difficult to guarantee a gas protection atmosphere. As a solution, we wrap the whole device with sealing films to protect it from water vapor in the air.

### IL gating of in-plane SAF heterostructures

**Fig. 4:**
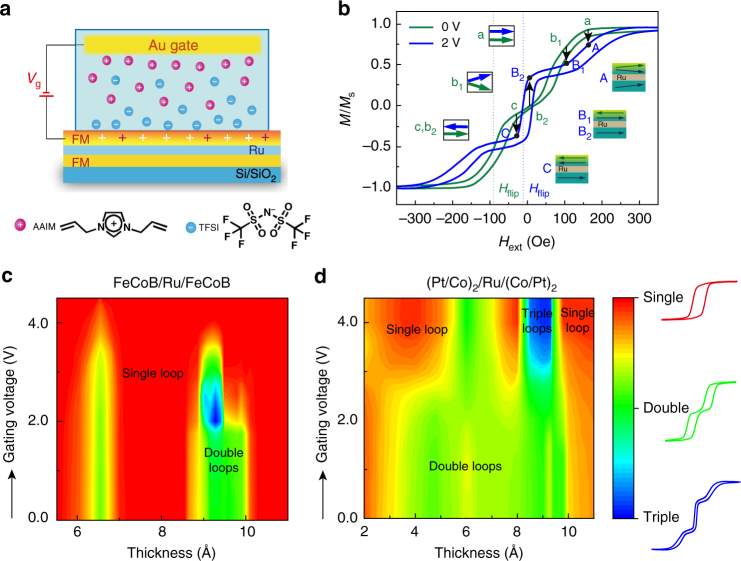
Discussion and generalization for voltage-controlled RKKY interaction. **a** Schematic of IL gating magnetism modification process for FM/Ru/FM SAF multilayers. **b** Hysteresis loops of magnetic moment switching during the transition of double-loop and triple-loop pattern. The IL gating effect for FeCoB (1.5 nm)/Ru (0.92 nm)/FeCoB (1.5 nm) SAF structure is chosen as a representative diagram. **c** In-plane and **d** out-of-plane RKKY interaction phase diagram, which summarize the variation of hysteresis loops with Ru thickness and gating voltage

First of all, we realized voltage control of RKKY interaction for in-plane configuration with FeCoB/Ru/FeCoB sandwich structure. When *t*_FeCoB_ = 4.5 nm, the IL gating process has a very small effect, which can be seen in Supplementary Fig. 3a. As demonstrated by Zhao et al., IL gating process only influences the interfacial layer no more than 3 nm thickness^32^. Thus, we refabricated SAF multilayers with a structure of FeCoB (1.5 nm)/Ru (*x* nm)/FeCoB (1.5 nm) and systematically studied the voltage control effect via IL gating process. The M-H loop variation shown in Fig. 2 clearly demonstrates a strong dependence on Ru thickness^27^. When *t*_Ru_ is around 0.65 nm, trilayer obtains a strong short-period oscillatory coupling^27^, but the magnetic hysteresis loops are barely changed with the applied *V*_g_ as shown in Fig. 2b. In contrast, as Ru thickness increases to about 0.95 nm, the strength of long-period oscillatory coupling becomes much weaker^23,27^, the applied *V*_g_ can have a dramatic effect on the SAF structures. As shown in Fig. 2d, e, when *V*_g_ = 2 V, the hysteresis loops change from initial double-loop to triple-loop pattern, from pure AFM coupling state to co-existence of AFM and FM coupling. If *V*_g_ keeps increasing to 4 V, hysteresis loops finally turn into single loops with only FM coupling. For the single area with thin spacer layer (Fig. 2c), the IL gating process is not that effective; this probably because that the surface modification becomes insensitive when the moments have strong parallel arrangements between the two FM sublayers^23,32^. The FM mode with a weaker IEC coupling can be affected by *V*_g_ as shown in Fig. 2f. We will further discuss this IL gating-induced magnetism modification in Fig. 4.

### IL gating of out-of-plane SAF heterostructures

For the out-of-plane (Pt/Co)_2_/Ru/(Co/Pt)_2_ SAFs, we minimized the number of lamination layers to two because the IL gating effect on (Pt/Co)_3_/Ru/(Co/Pt)_3_ is not obvious, as shown in Supplementary Fig. 3b. Perpendicular SAF multilayers fabricated here are (Pt 9 Å/Co 7.5 Å)_2_/Ru (*x* nm)/(Co 7.5 Å/Pt 9 Å)_2_. All the initial results of hysteresis loops shown in Fig. 3 confirm a *t*_Ru_ dependence on RKKY interaction, which is also displayed in Fig. 1e, f. As *t*_Ru_ increases from 0.2 to 1.1 nm, the strength of RKKY interaction first rises and then falls. For *t*_Ru_ = 0.2 nm (Fig. 3b), the spacer layer is discontinuous, thus PMA dominants in the competition with IEC. The RKKY interaction appears while under an applied *V*_g_ (red line in Fig. 3b) because of weakened PMA by IL gating process. And this decrement effect on PMA with positive *V*_g_ at film electrode has already been confirmed by Liu et al. recently (our positive *V*_g_ corresponds to their negative *V*_g_)^53^. As *t*_Ru_ keeps increasing, the competition between PMA and IEC becomes complex, thus the IL gating magnetism modification displays a dramatic and diversiform transition during these in-situ VSM measurements. When *t*_Ru_ = 0.4, 0.8 nm, hysteresis loops turn from double to single as shown in Fig. 3c, e. While at 0.6 and 0.94 nm, E-field-induced hysteresis loops reveal an enhanced RKKY interaction by exhibiting double-loop patterns in the farther distance (Fig. 3d, h). Similar to in-plane configuration, the triple-loop pattern is also observed at the critical thickness as displayed in Fig. 3f, g. But the applied voltage rises to 4 V in this system, which corresponds to a larger electrochemical window as shown in Supplementary Fig. 2b. As *t*_Ru_ increases to about 1 nm, RKKY interaction intensity gradually decreases and the IL gating process gives this IEC coupling a further weakening effect, maintaining a single hysteresis loop as displayed in Fig. 3i.

It is necessary to mention that when *V*_g_ is outside the electrochemical window (*V*_g_ ≧ 1.1 V for FeCoB, *V*_g_ ≧ 2.1 V for Co/Pt), there are ions of film plane coming into IL; film morphology may be changed and complex chemicals with Fe, Co, B ions (or Co, Pt ions) may form in IL^32^. Morphology analysis and changes of magnetic compositions are shown in Supplementary Figs. 4 and 5. This modification shows some kind of influences on the film properties, but interesting interfacial ME phenomena are observed under this complicated regulation as displayed in Figs. 2 and 3. To guarantee the reversibility of this IL gating modification, inactive nitrogen gas protection (very difficult for VSM) and top protecting layer (weakening the control effect) are necessary for some specific systems. We tried to improve the reversibility mainly based on ferromagnetic resonance (FMR) measurement, which has a closed cavity for nitrogen gas protection and can quantify the voltage-controlled resonance field shift with great precision of <1 Oe at the same time^32^. Top ultra-thin protecting layers are also necessary for the both SAF heterostructures. By making some compromises on the control effect, significant improvements of reversibility are realized as shown in Supplementary Fig. 6 (FeCoB system) and Figure 7 (Co/Pt system).

## Discussions

This IL gating-induced RKKY interaction modification is unclear, and we try to give a discussion for a preliminary understanding. Figure 4a gives a plane schematic for the charge accumulation and electrochemical reaction process. Red+ in FM layer illustrates the complex chemicals that may form in IL when *V*_g_ is outside the electrochemical window^32^. Magnetic moment switching in hysteresis loops have demonstrated a dramatic magnetism change under *V*_g_; the most interesting change is the transition between double-loop and triple-loop pattern. Details of the magnetization reversal can be understood from Fig. 4b, which shows relative angle variations of the two FeCoB layers as a function of external field. For the ungated state (green line), since the two FM layers are not exactly identical^28,54^, we use two different colors of arrows to distinguish two moments. During the IL gating process at *V*_g_ = 4 V (blue line in Fig. 4b), the electrochemical reaction happens in the SAF system changes film morphology and induces a third magnetization state at the surface (relative states are listed as A, B_1_, B_2_, C). Therefore, after IL gating, the SAF structure carries a totally different magnetization state at the same external field. Saturated parallel magnetic moments change to unsaturated canted states (a → A). Different initial magnetic moments (canted state for b_1_, antiparallel state for b_2_) can transfer into similar alignments (B_1_, B_2_); while the identical magnetic moments (c, b_2_) at the beginning may switch to two distinct (C, B_2_) alignments. It has been demonstrated that in the metallic systems with itinerant electron magnetism, the materials’ intrinsic magnetic properties are primarily determined by the surface electron density at the Fermi level^34^. Besides, whether the RKKY interaction is FM mode or AFM has a strong correlation with iterant electrons and the thickness of NM layer^27^. Since *t*_Ru_ is fixed for every single sample and there is no potential lattice distortion, the response of iterant electrons becomes the dominant factor when the surface charges accumulate. Therefore, we attribute this transition phenomenon to the disturbance of iterant electrons inside SAF heterostructures and the corresponding change of Fermi level, which is due to the enormous interfacial charge accumulation aroused by IL gating process.

A simple model has been theoretically analyzed with an interpretation for the magnetic reversal of antiparallel coupled systems^46^. The AFM coupling strength of original green curve shown in Fig. 4b can be calculated approximately by the following relation: ${J} = - \left( {{M}_1{t}_1 + {M}_2{t}_2} \right){H}_{{\mathrm{flip}}}$, where *t*_1_ and *t*_2_ are thickness of FM layers and *M*_1_, *M*_2_ are the relevant saturation magnetizations (*M*_1_ = *M*_2_ = 1100 emu cm^−3^), *H*_flip_ in the figure refers to the characteristic spin-flip field for AFM mode (94 Oe)^28,46^. Spin-flip field refers to the sharp switch field where magnetization reversal is the swiftest^46^. Therefore, we obtain the typical AFM coupling strength with the value of 0.03 erg cm^−2^. As for the coupling strength of gated blue curve, two spin-flip fields should be considered and we propose a relation as ${J} = - {{\rm Mt}}{\kern 1pt} {H}_{{\mathrm{flip}}}$ for approximate evaluation. The saturation value and thickness of three magnetization states are hard to decide separately, so we assume the product of Mt remain the same and obtain the value of 0.004 erg cm^−2^. Although the inner mechanism here remains unclear and needs further exploration, this way of electrically manipulated spin ordering will definitely shed light on the new generation of GMR ME memory prototype structure with advanced functionality.

The hysteresis loops variation with Ru thickness and *V*_g_ discussed in Figs. 2 and 3 are summarized as phase diagrams, which can be found in Fig. 4c, d. The red, green, and blue regions represent single, double, and triple loops, respectively. Different color depths are used in these two figures according to the existing experimental data as well as estimations of IEC status, which give a qualitative view of the gating effect on RKKY interaction. The quantitative determination of IEC coupling can be carried out through in-situ FMR measurement as shown in Supplementary Fig. 8. We calculated both AFM and FM coupling strength of perpendicular SAF at various *t*_Ru_ and determined the changes of effective RKKY interaction (J_RKKY_) after IL gating. These results provide a quantitative evidence for voltage control of RKKY interaction.

### Voltage control of dynamic domain switching for out-of-plane configuration

**Fig. 5:**
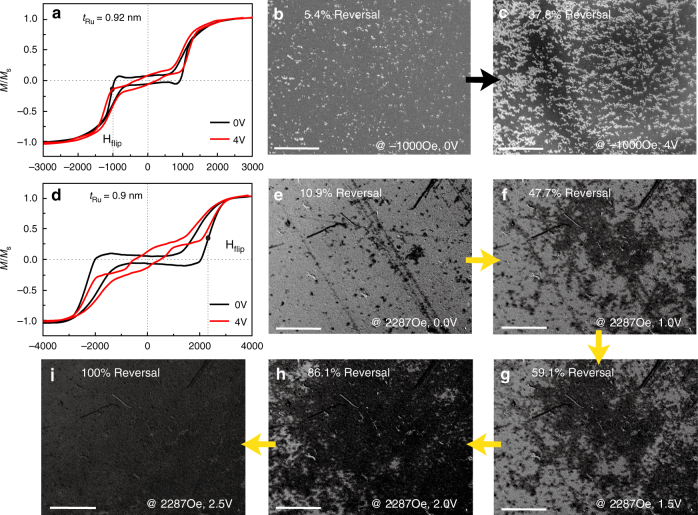
Voltage control domain switching evidenced by (Pt/Co)_2_/Ru/(Co/Pt)_2_/glass SAF heterostructures. **a** Ex situ VSM hysteresis loops at *t*_Ru_ = 0.92 nm. **b**,**c** are corresponding domain nucleations at 0 and 4 V. The voltage dwell time is 5 min. **d** Ex situ VSM hysteresis loops at *t*_Ru_ = 0.9 nm. **e**–**i** are the corresponding domain switching images at various *V*_g_. The determination of reversal ratio: count the ratio of light (**b**, **c**) and shade (**e**–**i**) at first, and then normalized by the value of saturated image

Voltage control of perpendicular magnetic moments reversal was also studied through in-situ Kerr microscopy images based on (Pt 9 Å/Co 7.5 Å)_2_/Ru/(Co 7.5 Å/Pt 9 Å)_2_ multilayers on glass substrates. Here, transparent substrate was chosen to let the light penetrate from the back of the substrate. We put sample upside down on the IL and a planar electrode served as both the conductive side and a bottom tray. Since the IL was also transparent, we managed to observe the voltage modification at exactly the same area of IL/film interface. Figure 5 shows the domain evolutions during IL gating process. Our samples are found to be non-volatile. The test performance is not changed at any time after the voltage is removed. Thus the ex situ measurement is acceptable. Figure 5a–c are results at *t*_Ru_ = 0.92 nm. Figure 5a shows the related ex situ hysteresis loops measured by VSM serving as a reference. We saturated samples with a large positive H-field, then decreased H-field until it reached the negative spin-flip field (−1000 Oe), where domain switching was most active. As shown in Fig. 5b, before E-field is applied, only 5.4% domain rotates; when *V*_g_ = 4 V, the reversal ratio gradually increases to 37.8% (Fig. 5c). Then we utilized another sample with *t*_Ru_ = 0.9 nm to study how domain evolves along the *V*_g_ variation. Figure 5d is the related hysteresis loops. In this case, we let H-field start at the negative saturation field and remain at +2287 Oe (H_flip_). When *V*_g_ gradually increases from 0 to 2.5 V, domain reversal ratio changes from 10.9 to 100% and keeps the same despite further *V*_g_ increasing. These phenomena indicate that up to 80% of perpendicular magnetic moments manage to switch with a small-applied voltage of 2.5 V. Details can be found in Supplementary Movie 1 Therefore, *V*_g_ has a significant influence on domain switching process and can control magnetization reversal effectively. Besides, the relationship of domain evolutions along with H-field can also be controlled by *V*_g_ as demonstrated in Supplementary Fig. 9. Nucleation field dramatically changes ~1300 Oe after gating at 4 V, confirming that IL gating process is promising in domain regulations. This achievement is promising in the voltage tunable RKKY memories with ultrahigh storage density.

### First principle calculations of voltage-induced IEC change

To theoretically understand the gating effect on the IEC, we use a Green function method with TB-LMTO^55^ to calculate the IEC from the first principle as:

1 $$J\left( \theta \right) = - \frac{1}{\pi }{\mathrm{Im}}{\int}_{\!\!\!\!\mathrm{\Lambda }} {{\int}_{\!\!\!\!C}\ {f\left( z \right){\mathrm{tr}}{\kern 1pt} {\mathrm{ln}}\left[ {1 - \frac{{1 - {\mathrm{cos}}{\kern 1pt} \theta }}{2}M\left( {k_\parallel ,z} \right)} \right]{\rm d}z} },$$

where *θ* is the angle between two FM layers, *f*(*z*) is the Fermi-Dirac distribution for a complex energy *z*, *Λ* is the total lateral 2D Brillouin zone (BZ) area with sampled 20,000 *k*_||_ inside to make sure the results converged in this work and *M*(*k*_||_, z) can be further expanded as:

2 $$\begin{array}{*{20}{l}} {M\left( {k_\parallel ,z} \right)} \hfill & = \hfill & { - \left( {1 - S_{\rm RL}^\alpha \overline {{\cal G}_{\rm L}^ \uparrow } S_{\rm LR}^\alpha \overline {{\cal G}_{\rm R}^ \uparrow } } \right)^{ - 1}S_{\rm RL}^\alpha \left( {\overline {{\cal G}_{\rm L}^ \uparrow } - \overline {{\cal G}_{\rm L}^ \downarrow } } \right)} \hfill \\ {} \hfill & {} \hfill & {\left( {1 - S_{\rm LR}^\alpha \overline {{\cal G}_{\rm R}^ \downarrow } S_{\rm RL}^\alpha \overline {{\cal G}_{\rm L}^ \downarrow } } \right)^{ - 1}S_{\rm LR}^\alpha \left( {\overline {{\cal G}_{\rm R}^ \uparrow } - \overline {{\cal G}_{\rm R}^ \downarrow } } \right)} \hfill \end{array}$$

To use the surface Green function (SGF) technique, the device is divided into the left (L) and right (R) subsystems by the middle of the Ru, $S_{\rm RL/LR}^\alpha$ denotes the coupling of the neighboring principle layers between the left and right subsystems, and $\overline {{\cal G}_{\rm L/R}^\sigma }$ are the configurational averaged SGF of the left/right part for the spin *σ*^56^. To compute the averaged SGF, the coherent-potential approximation^57–59^ is applied in combination with the renormalization-decimation technique^60^.

**Fig. 6:**
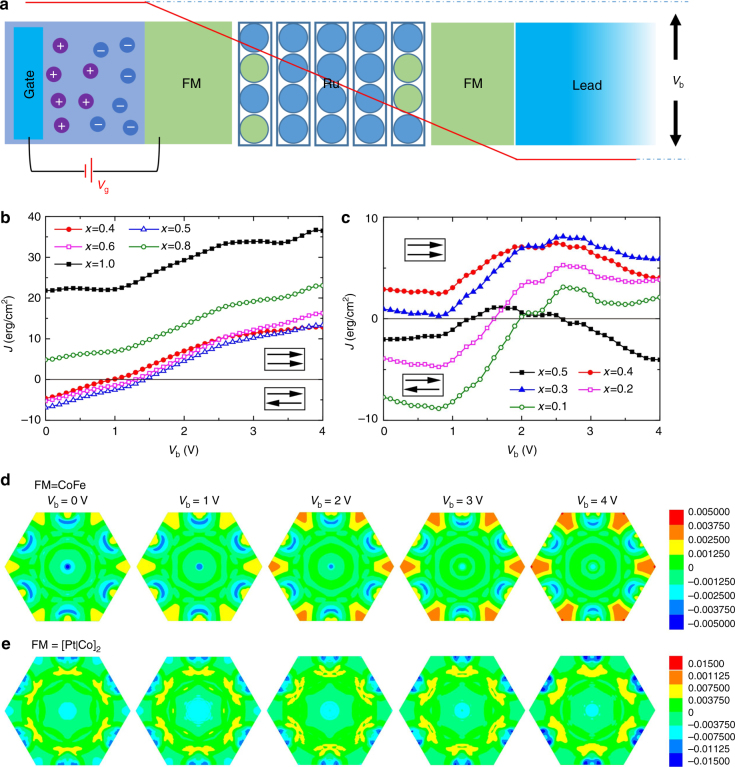
Interlayer exchange coupling (IEC) simulation for Lead/FM/Ru/FM/Lead structure. **a** Schematic of the simulation model, here we chose Cu as a common lead and FM represents CoFe alloy or Pt|Co multilayers. Basically, the Ru on both left and right interfaces can be permeated by FM atoms as interface disordering. Under an applied *V*_g_, the chemical potential (Fermi energy) of the contacted FM layer was shifted up, which generated an equivalent voltage on the spin valve as $V_{\mathrm{b}} \approx V_{\mathrm{g}}$. The red line stand for the artificial potential drop with *V*_b_. **b**, **c** represent the IEC of FM = CoFe (1.5 nm) with 5 atomic layer of Ru (~1.1 nm) inside and FM = [Pt(0.88 nm)|Co(0.70 nm)]_2_ with 4 atomic layer of Ru (~0.88 nm) inside, respectively. We calculated the IEC as a function of *V*_b_ and interface disorder concentrations *x*, which means that the interface is structured as Ru_*x*_(CoFe)_1-*x*_ or Ru_*x*_Co_1-*x*_ for different cases. **d**,** e** stand for the dimensionless *k*_||_-resolved IEC for FM = CoFe and FM = [Pt|Co]_2_ multilayer, respectively. Here we chose five *V*_b_ points from **b** (*x* = 0.5) and **c** (*x* = 0.1) under various gating voltages

Nevertheless, it is easy to know that *J*(*θ*) reach maximum value when *θ* = *π* and other values can be obtained analytically, so we use *θ* = *π* for IEC calculations in room temperature (*T* = 300 K) with the structures as shown in Fig. 6a. The voltage *V*_b_ stands for the potential shifting during IL gating (red line), which can be treated as similar as *V*_g_, since they both shift the band filling of contacted FM layer equivalently. The IEC results with various Ru thicknesses are summarized in Fig. 6b, c.

For FM = CoFe in Fig. 6b, when the interfaces are clean (*x* = 1.0), the IEC of the system shows a FM coupling and it increases with increasing gating voltage. While introducing an interface disorder (*x*), the effective Ru thickness ($t_{{\mathrm{Ru}}} = 0.22\left( {n - 2} \right) + 2x \ast 0.22$, where n is the atomic layer of Ru) was reduced. The IEC becomes smaller and results in a cross point with zero level at about 1.5 V. In this case, the system can be switched from an AFM coupling to FM coupling by increasing the *V*_g_ at certain Ru thickness range (0.84 ~ 0.92 nm), which agrees well with the experiments (0.88 ~ 1 nm).

For FM = [Pt(0.88 nm)|Co(0.70 nm)]_2_ multilayers in Fig. 6c, we can also obtain similar conclusion as that of FM = CoFe. Here, the changing from AFM coupling to FM coupling appears in low *x* (*x* = 0.1, 0.2) at about 2 V, referring to a Ru thickness of (0.48–0.53 nm). While *x* increases to 0.5 (*t*_Ru_ = 0.66 nm), there is a relatively weak AFM coupling at the initial state and can be switched into a strong AFM coupling at *V*_g_ = 4 V, corresponding to the yellow region around 0.6 nm demonstrated in Fig. 4d. By summarizing RKKY interaction strength of Fig. 4c, d, we can find similar features between the simulations and experiments.

Figure 6d, e stand for the dimensionless *k*_||_-resolved IEC for FM = CoFe and FM = [Pt|Co]_2_ multilayers, respectively. For the FM = CoFe case, it is obvious that even without applied voltage (*V*_b_ = 0 V) the *k*_||_-resolved IEC could be either positive or negative. However, the integration shows a negative value in total, which gives an AFM coupling. With increasing the applied voltage, the positive zones (warm colar, FM coupling) become larger and their values increase; while the negative zones (cold color) become smaller. These texture changes end up with an IEC sign switch under the applied gating voltage. Similarly, we can also observe the identical behavior with FM = [Pt|Co]_2_ multilayer in Fig. 6e, which means that the physical origin of the gating effect for FM = CoFe and FM = [Pt|Co]_2_ multilayers are similar. As thus, based on these analyses, the IL gating process is able to change the strength of IEC by changing the band filling of FM (Fermi energy). Additionally, the IEC can vary from AFM coupling to FM coupling with some interface disorders under various voltages, which is quite similar as that in MTJs^55^.

However, it is hard to define a same thickness between the theoretical model and experiments, as that the interface disorder between Ru and FM may remarkably change the thickness of Ru in the scale of angstrom. And the phase diagram in Fig. 4 shows a rich behavior in the scale of angstrom, while the first principle calculations in Fig. 6 is also sensitive to the interface disorder. Although there exists a gap when one tries to match the theoretical and experimental results, we declare an efficient way to handle the switching of magnetism by IL gating. This proceeding method is the most important part and has been strongly supported by both experiment and first principle.

In summary, we demonstrated E-field manipulation of RKKY interaction based on Au/[AAIM]^+^[TFSI]^−^/FM/NM/FM SAF structures on SiO_2_/Si and glass substrates. First of all, switches of single, double, and triple pattern were achieved in both in-plane and out-of-plane systems, corresponding to the different extent of AFM or FM coupling. Secondly, voltage control of perpendicular domain switching was demonstrated and IL gating process proved to be effective in the regulation of dynamic magnetization reversal. Besides, E-field-induced variation of AFM coupling strength is calculated in both qualitatively and quantitatively way to interpret the typical magnetic reversal. Theoretical simulation of the voltage-induced RKKY interaction process was first investigated and confirmed our experimental results perfectly.

From the device perspective, IL has potential problems with leaking and pollution. As an alternative, IL gel can be more stable and convenient than IL itself^61,62^, which can be studied further in the future. What’s more, if AFM-coupled SAF structure is constructed with an extremely high precision, there will be domain nucleations at 0 Oe when the spin-flop transition happens. This provides a promising possibility for the voltage control of magnetization switching without a bias magnetic field. All in all, our work provides a platform to manipulate AFM spintronics by interfacial ME effect and bridges the FM and AFM spintronics through E-field modulation. This E-field assisted moment switching in hysteresis loops will enable the realization of power efficient AFM MRAMs and novel GMR devices with ultrahigh densities.

## Methods

### Film growth

Fe_40_Co_40_B_20_ (1.5 nm)/Ru (*x* nm)/Fe_40_Co_40_B_20_ (1.5 nm)/Ta (7.5 nm) and (Pt 9 Å/Co 7.5 Å)_2_/Ru (*y* nm)/(Co 7.5 Å/Pt 9 Å)_2_/Ta (3.5 nm) multilayers with different Ru thickness (*x*, *y*) were deposited onto SiO_2_/Si and glass substrates at room temperature. The base pressure is less than 10 Torr and the working Ar pressure is 3 mTorr with a DC power of 20–30 W.

### Device fabrication

Gold wires were used as gate electrode and connecting line between surface film plane (i.e., FeCoB, Pt) and B2901A Electrometer. The IL [AAIM]^+^[TFSI]^−^ was dropped onto the film plane directly contacting two electrodes. More device details were shown in the Figs. 2a and 3a. Dry nitrogen gas protection is guaranteed in FMR measurements for there was an annular seal chamber. During the VSM measurement, sealing film was utilized as a compromise for the test difficulty.

### TEM characterization

The microstructure of the film was characterized by HR-TEM using JEM-2100.

### Magnetic properties characterization

In-situ IL gating was carried out in VSM (Lake Shore 7404), MOKE microscope (Evico Magnetics, em-Kerr-Highres), and FMR (JEOL, JES-FA200) measurements. For the domain observation carried by MOKE microscope, we used the polar MOKE mode with perpendicular electromagnet. The gating voltage was applied by Agilent B2901A electrometer. During all the IL gating process, we waited 5 min (±15 s) before formal measurements to ensure the charge balance. All the characterizations are performed at room temperature.

### Data availability

The relevant data from this study are available from the authors.

## Electronic supplementary material

Supplementary Information

Description of Additional Supplementary Files

Supplementary Movie 1
