# Supplementary material for: Ionic liquid gating control of RKKY interaction in FeCoB/Ru/FeCoB and (Pt/Co)2/Ru/(Co/Pt)2 multilayers
Source: Nat Commun. 2018 Mar 7;9:991. doi: 10.1038/s41467-018-03356-z (PMC5841336; doi:10.1038/s41467-018-03356-z)
Supplement: Supplementary file 1 — Supplementary Information [file 41467_2018_3356_MOESM1_ESM.docx]

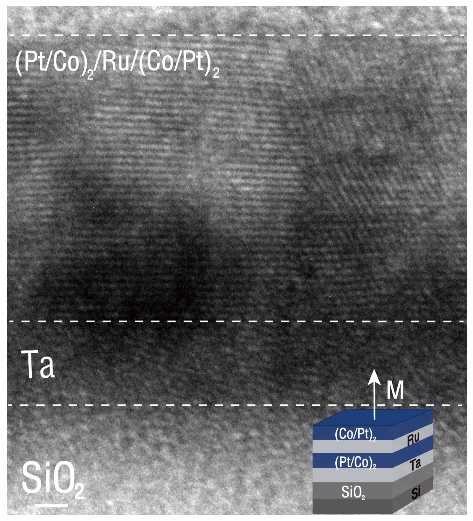


**Supplementary Figure 1 | High-resolution image for Co/Pt polycrystalline structure.** High-resolution image of (Pt 9 Å/Co 7.5 Å)_2_/Ru (0.95 nm)/(Co 7.5 Å/Pt 9 Å)_2_/Ta (3.5 nm)/SiO_2_/Si examined by HR-TEM. The staggered lines indicated the polycrystalline structure of this perpendicular SAF. Scale bar, 1 nm.

**Supplementary Note 1. Test of electrochemical window based on the VSM measurement condition.**

The [AAIM]^+^[TFSI]^-^ electrochemical windows were test with B2901A Precision Source/Measure Unit. The sweep loops were processed with a slow sweeping rate (6.25 mV s^-1) to reduce the current induced by ionic migration[^1^](#_ENREF_1). Research has found that *V*_g_ window range is varying with material systems and is not affected by film thicknesses[^1^](#_ENREF_1). Therefore, we believe these two *V*_g_ window ranges are suitable for all our samples. As shown in Supplementary Figure 2, the range of the gating voltage was divided into two regions, region I (−1.7 V < *V*_g_ < 1.1 V for FeCoB, −2 V < *V*_g_ <2.1 V for Co/Pt) and region II (*V*_g_ ≧1.1 V and *V*_g_≦-1.7 V for FeCoB, *V*_g_≧2.1 V and *V*_g_≦-2 V for Co/Pt). In region II, there is a clear current increase, accompanying by complex chemical reactions. We utilized this region and obtain interesting interfacial effect. For the sake of simplification, only positive voltage of region II is considered in this study, where current is increasing relatively stable compare that in the negative voltage region.


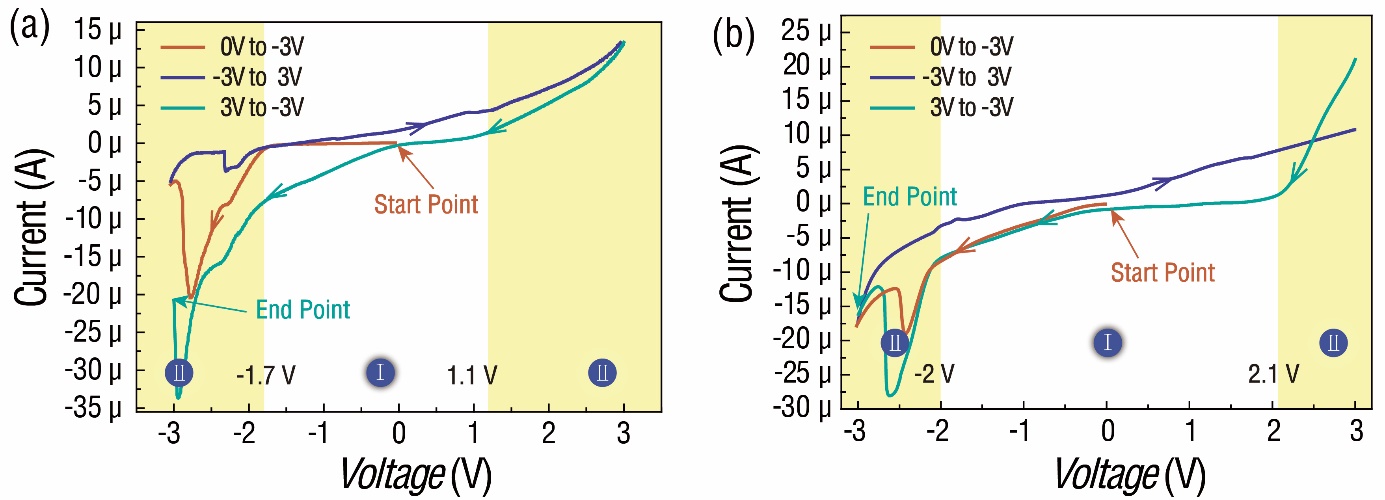


**Supplementary Figure 2 | Electrochemical window tests of the in-plane and out-of-plane SAFs.** (a) Electrochemical window for FeCoB (1.5 nm)/Ru (0.9 nm)/FeCoB (1.5 nm)/Ta (7.5 nm)/SiO_2_/Si. Au and FeCoB (1.5 nm) serve as electrodes. (a) Electrochemical window for (Pt 9 Å/Co 7.5 Å)_2_/Ru (0.9 nm)/(Co 7.5 Å /Pt 9 Å)_2_/Ta (3.5 nm)/SiO_2_/Si. Au and Pt (9 Å) serve as electrodes.

**Supplementary Note 2. Test for proper film thickness and structure to maximize the voltage controlled effect**

First of all, we tested the *t*_FeCoB_ for FeCoB/Ru/FeCoB system to maximize this surface modification effect. While varying *t*_Ru_ within 1 nm, AFM mode (double S shape hysteresis loop) only appeared around 0.6 nm when *t*_FeCoB_ = 4.5 nm, and the IL gating process have a very small effect on this area. Typical control result can be seen in Supplementary Figure 3(a). Then we decrease *t*_FeCoB_ to 1.5 nm, the voltage controlled IL-gating effect becomes obvious as displayed in Figure 2 of the main text.

For (Pt/Co)*_n_*/Ru/(Co/Pt)*_n_* system, we mainly tested the number of laminations (i.e., *n*). The larger the value of n, the stronger the PMA effect. Since the magnetic behavior of perpendicular SAF multilayers is a synthesis of both IEC and PMA, we minimized the number of laminations to two layers to weaken PMA and strengthen RKKY interaction. This way works well as demonstrated in Supplementary Figure 3(b) and Figure 3. IL gating effect on (Pt/Co)_3_/Ru/(Co/Pt)_3_ is far weaker than that of (Pt/Co)_2_/Ru/(Co/Pt)_2_.

**
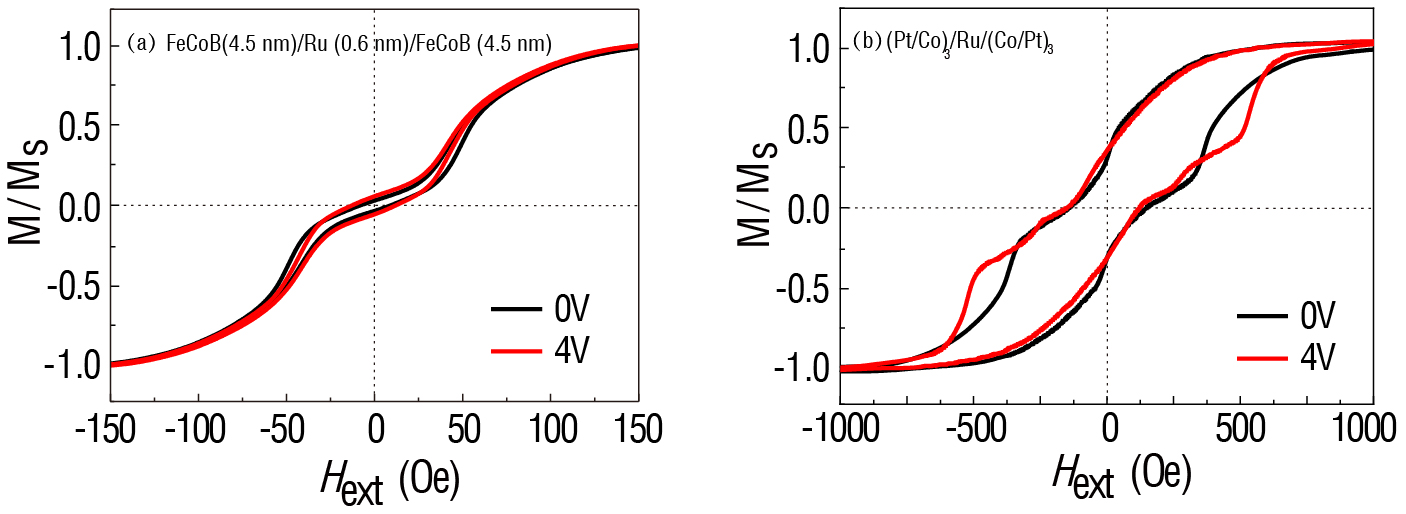
**

**Supplementary Figure 3 |** **In-situ VSM test for in-plane and out-of-plane configuration while under IL-gating magnetism modification.** SAF structures here are FeCoB (4.5 nm)/Ru (0.6 nm)/FeCoB (4.5 nm) for (a) and (Pt 9 Å/Co 7.5 Å)_2_/Ru (8.8 Å)/(Co 7.5 Å /Pt 9 Å)_2_ for (b). The applying magnetic field was along easy axis of each system.

**Supplementary Note 3. Morphology analysis before and after gating.**

Supplementary Figure 4 displays typical AFM images of the film surfaces before and after gating process. Ungated films carry smooth surfaces as displayed in Supplementary Figure 4(a) and (d). After gating at 4 V, both rough and smooth areas are obtained (Supplementary Figure 4(b), (c) for FeCoB; Supplementary Figure 4 (e), (f) for Co/Pt), indicating this IL gating process at high voltage obtains uneven effects. Samples for Supplementary Figure 4(b), (c) and Supplementary Figure 4(e), (f) are the same respectively, but with different test areas. Through the morphology changes, existences of electrochemical reactions are confirmed. Since the effect is highly depended on different locations, in this case, just looking at a single point may take a part for the whole.


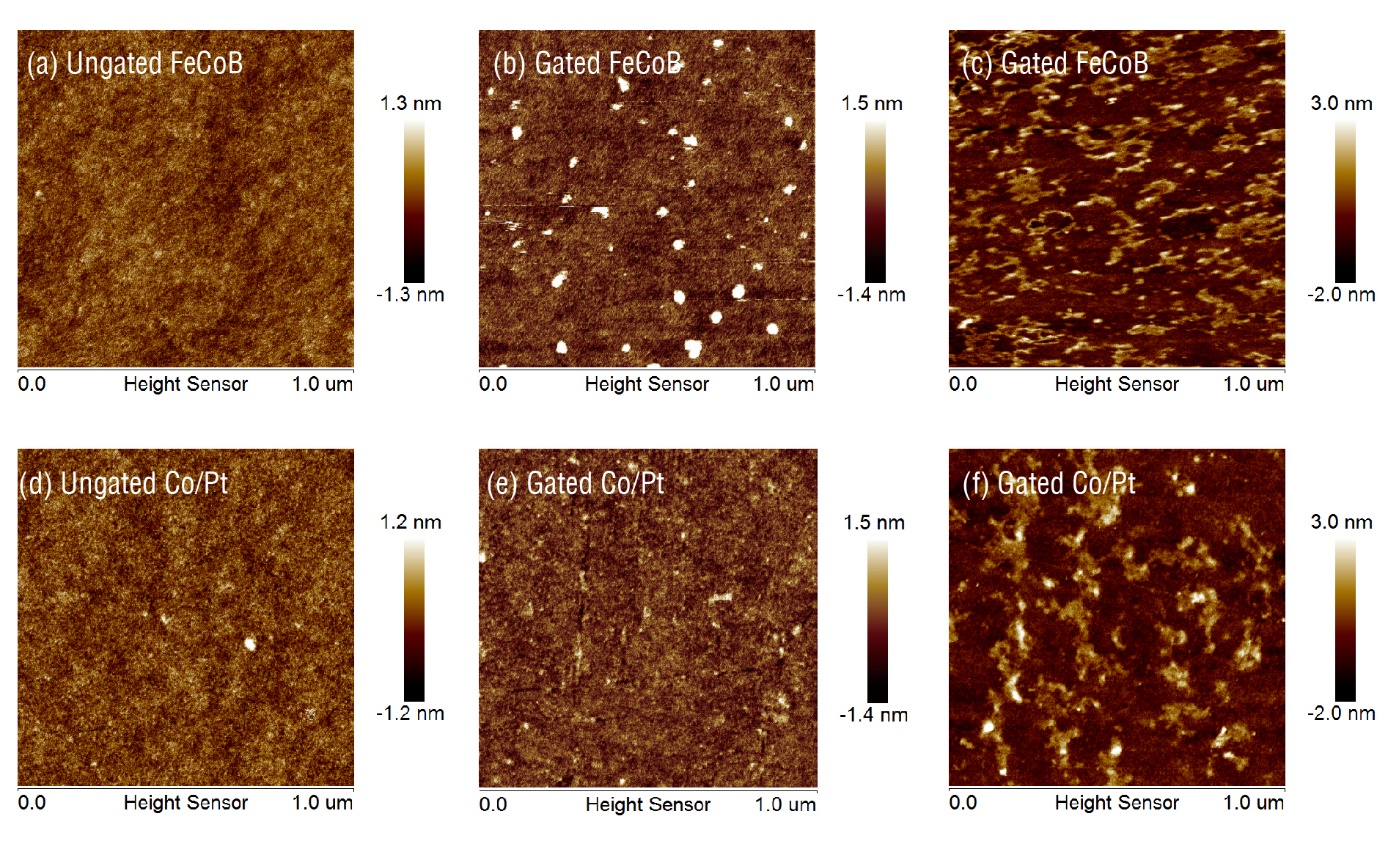


**Supplementary Figure 4 | AFM for in-plane configuration and out-of-plane configuration.** (a) AFM measurement of FeCoB surface for FeCoB (1.5 nm)/Ru (0.9 nm)/FeCoB (1.5 nm)/Ta (7.5 nm)/SiO_2_/Si SAF multilayer before gating; (b, c) Ex-AFM of the FeCoB surface after gating at 4 V. (d) AFM measurement of Co/Pt surface for Pt 9 Å/Co 7.5 Å)_2_/Ru (0.94 nm)/(Co 7.5 Å/Pt 9 Å)_2_/Ta (3.5 nm)/SiO_2_/Si SAF multilayer before gating; (e, f) Ex-AFM of the Co/Pt surface after gating at 4V. In order to better compare the changes before and after gating, we use same samples in (a), (b), (c) and (d), (e), (f). Two samples were both processed at 4 V for 5 min, then the electrodes were removed and samples were washed with the alcohol.

**Supplementary Note 4. Changes of magnetic compositions analyzed by VSM and XRD**

We can see the change of magnetic compositions after gating at 4 V through Supplementary Figure 5. Supplementary Figure 5(a) is a typical un-normalized result and it shows that at higher *V*_g_, the intensity of hysteresis loop has a clear decline. XRD experiments were also performed to investigate how the gating process affects crystal structure. No obvious change has been observed except the peak intensity of Co as shown in Supplementary Figure 5(b) and (c). *V*_g_ outside electrochemical window may have changed the magnetic properties of films to some extent, but from the overall point of view, this is change is not a devastating disaster for thin films.

**
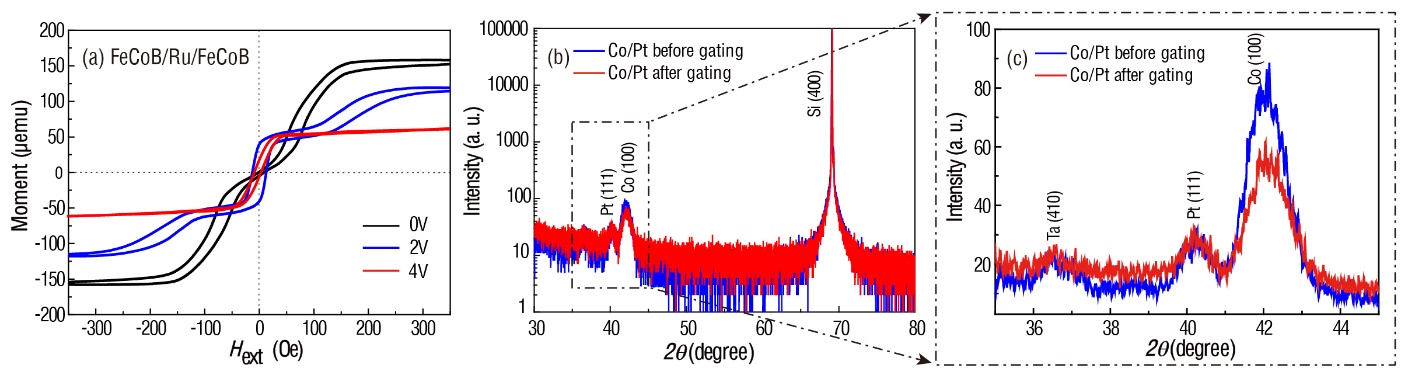
**

**Supplementary Figure 5 |** **Studies of magnetic compositions changes at different *V*_g_.** (a) In-situ VSM measurement for FeCoB (1.5 nm)/Ru (0.92 nm)/FeCoB (1.5 nm)/Ta (7.5 nm)/SiO_2_/Si SAF multilayer without data normalization. (b) Ex-XRD for (Pt 9 Å/Co 7.5 Å)_2_/Ru (0.98 nm)/(Co 7.5 Å /Pt 9 Å)_2_/Ta (3.5 nm)/SiO_2_/Si SAF multilayer before and after gating at 4 V. (c) shows the enlarged picture for (b). The ex-AFM and XRD measurements for gated SAF multilayers were all processed at 4 V for 5 min, then the electrodes were removed and samples were washed with the alcohol.

**Supplementary Note 5. Improvement of reversibility for FeCoB/Ru/FeCoB SAF multilayer**

The reversibility can be significantly improved by adding an ultra-thin protecting layer at the top of structure. As mentioned in the main text, it is difficult for VSM to have a gas protection atmosphere. Therefore, the reversibility study mainly based on FMR measurement, which has a closed cavity for nitrogen gas protection.

For FeCoB/Ru/FeCoB SAF multilayer, we utilized ~1 nm Ta cap layer to protect FeCoB from reacting when *V*_g_=4 V. The FM→AFM→FM transition can also be observed by increasing Ru thickness gradually, indicating that the RKKY interaction with an additive Ta cap player is comparable with the pure FeCoB/Ru/FeCoB system. Supplementary Figure 6(a) shows the typical double hysteresis loops for AFM coupled SAF structures. Supplementary Figure 6(b) is the corresponding FMR spectra for 0.95 nm Ru, 1.25 nm Ta sample (analysis for optical and acoustic modes is discussed in detailed in the part 8 of SI). Since the signal for OM mode is much stronger than AM mode, we mainly focused on the response of OM mode during IL-gating progress to guarantee the accuracy. We then studied IL-gating processes for the two samples. Supplementary Figure 6(c) are the FMR spectra for SAF structure with 1 nm Ta cap layer. The resonance field shift is ~20 Oe at *V*_g_=4 V. When V_g_ is switched off, the resonance field can come back ~5 Oe. Supplementary Figure 6(d) clearly illustrates that resonance field or the IL-gating modification can be switched back and forth with an alternating bias polarity, indicating a voltage-induced reversible control[^1^](#_ENREF_1). Supplementary Figure 6(e) is the results for 1.25 nm Ta capped sample. The control effect is weakened as Ta layer increases, but the recovery rate raises to ~50%. By alternating bias polarity between ±4 V, the control effect can be enhanced effectively. As shown in Supplementary Figure 6(f), reversible switching happens between 720 Oe and 780 Oe. These results indicate that protecting payer and gas protection are important for the reversibility of FeCoB system.


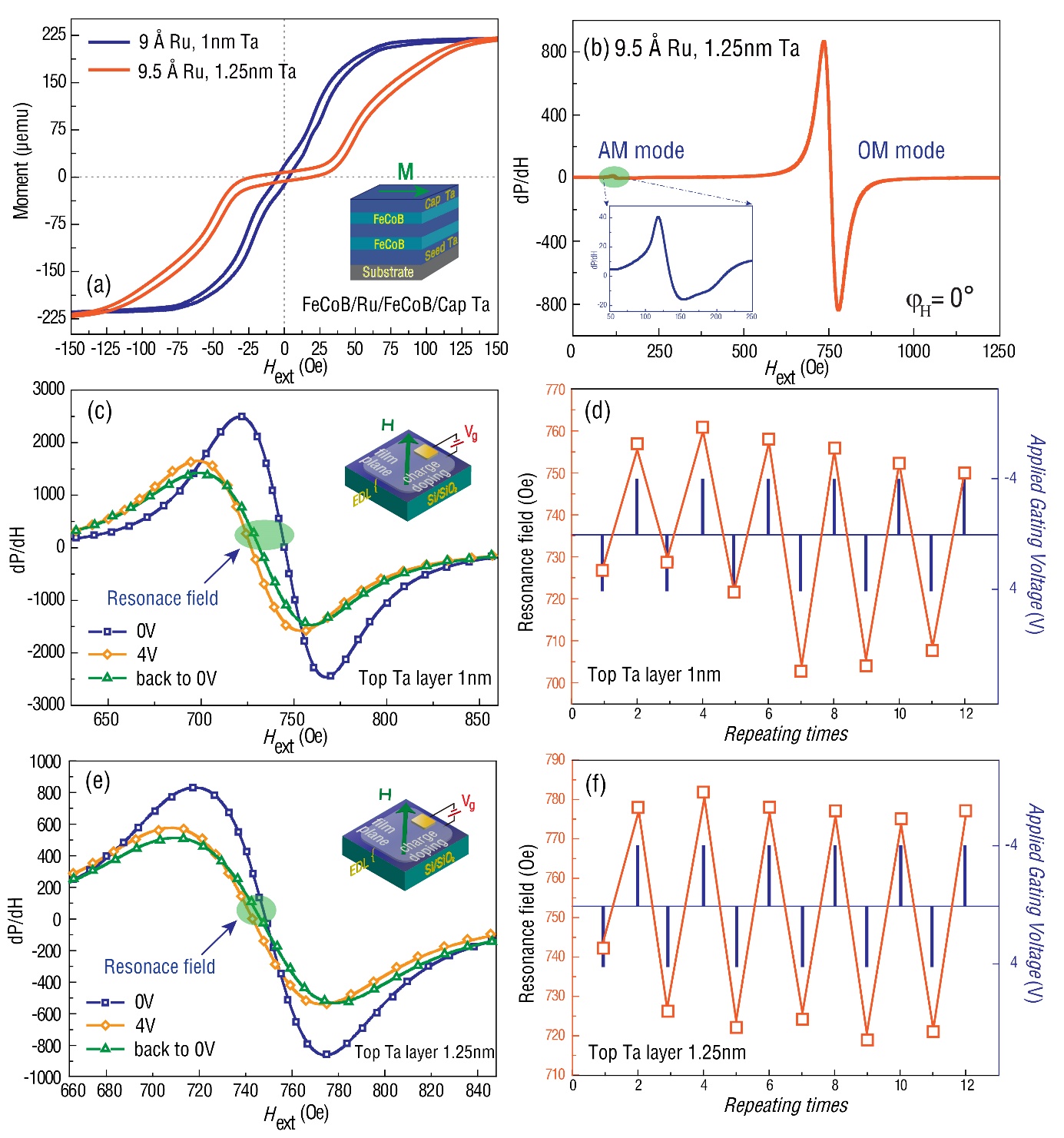


**Supplementary Figure 6 |** **Basic magnetic properties and reversibility studies for cap Ta/FeCoB/Ru (~1 nm) /FeCoB/seed Ta SAF multilayers with typical AFM coupling.** (a) Magnetic hysteresis loops for SAF structure with 1 nm and 1.25 nm Ta cap layer. (b) FMR spectra at $\varphi_{H}=0^{^{\circ}}$ (in-plane direction) for the strongly AFM-coupled multilayer at *t*_Ru_=0.95 nm, top*_Ta_*=1.25 nm. (c) In-situ FMR measurement for the sample at *t*_Ru_=0.9 nm, top*_Ta_*=1 nm, while (d) is the corresponding FMR field switching during repeated ±4 V voltage pulse. (e), (f) are the FMR spectra and reversible switching for 0.95 nm Ru, 1.25 nm Ta sample, respectively.

**Supplementary Note 6. Improvement of reversibility for (Pt/Co)_2_/Ru/(Co/Pt)_2_ SAF multilayers**

For Co/Pt system, the top Pt layer is a part of SAF structure but can also be used as a protecting layer. Significant improvements of reversibility can be obtained if we make some compromises on the control effect. When the top Pt layer is 1 nm, as shown in Supplementary Figure 6(a-c), the control effect is strong while the reversibility is quite slight. If top Pt thickness increases to 1.25 nm, clear IL-gating modification and good reversibility can be acquired at the same time (Supplementary Figure 6(d-f)). As Pt thickness of the top layer increases to 1.5 nm, the voltage-induced effect is a little bit slight while the reversibility becomes strong (Supplementary Figure 6(g-i)). These results indicate that proper adjustment of top Pt layer thickness is important for the improvement of reversibility in Co/Pt system. Besides, the hysteresis loops for the samples with modified Pt layer (Supplementary Figure 6(a), (d) and (g)) reveals a clear *t*_Ru_ dependence, which is consistent with the initial results shown in Figure 1(g). This declares that the RKKY interaction here is comparable with the original RKKY interaction.


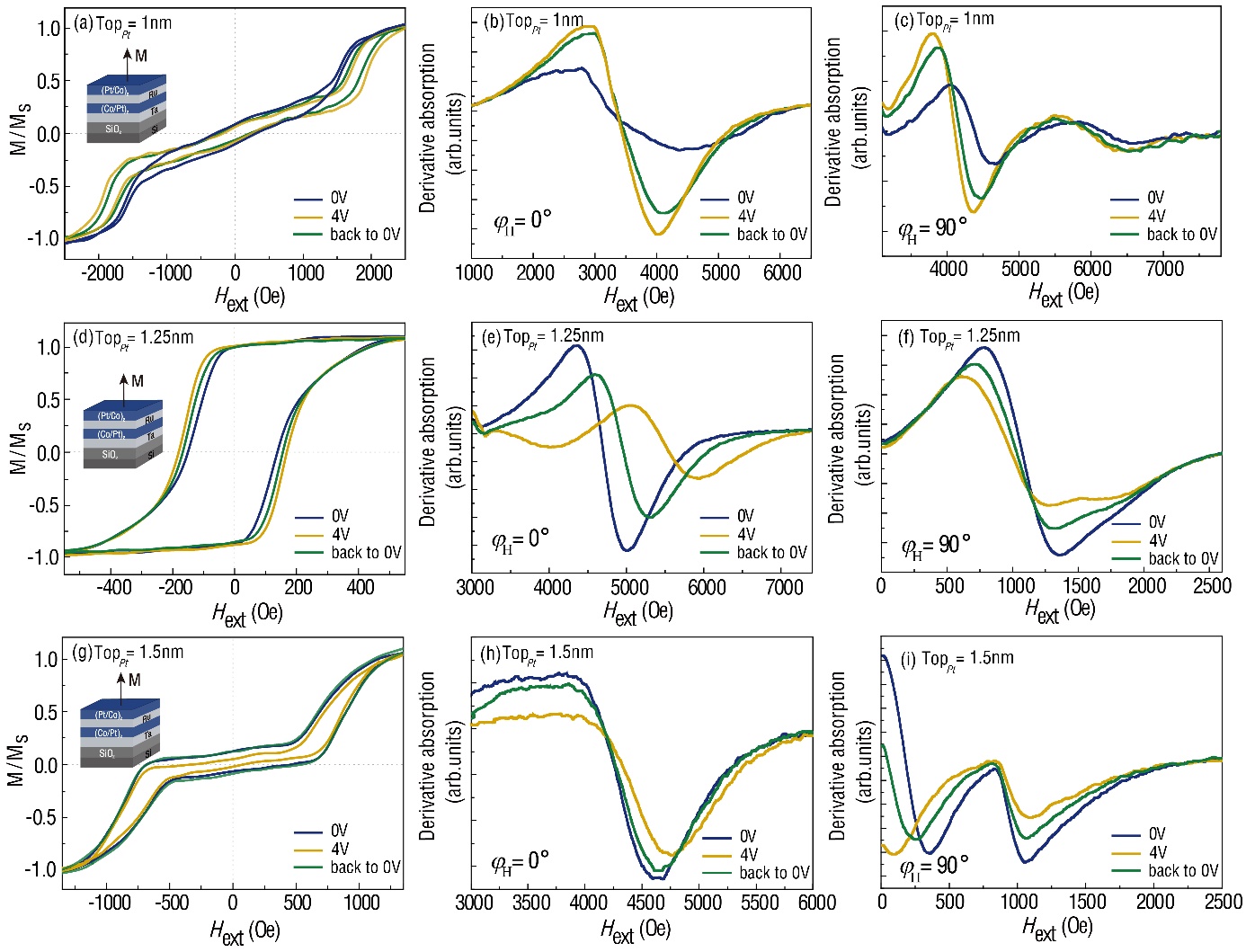


**Supplementary Figure 7 |** **Reversibility study for (Pt/Co)_2_/Ru/(Co/Pt)_2_ SAF multilayers.** (a) In-situ VSM and (b) in-plane, (c) out-of-plane FMR measurements for the sample with 0.9 nm Ru spacer and 1nm Pt cap layer. (d-e) are the results for *t*_Ru_=1nm, top*_Pt_*=1.25nm, while (g-i) are the results for *t*_Ru_=0.95nm, top*_Pt_*=1.5nm. $\varphi_{H}=0^{^{\circ}}$represents in-plane direction while ${90}^{^{\circ}}$ means out-of-plane direction.

**Supplementary Note 7. Calculation of effective RKKY interaction for (Pt/Co)_2_/Ru/(Co/Pt)_2_ heterostructure.**

To further confirm the IL gating effect on RKKY interaction and quantitatively determine the anisotropy change, we carried out in-situ spin dynamic measurement via FMR technique for perpendicular SAF (Pt/Co)_2_/Ru/(Co/Pt)_2_ heterostructures as displayed in Supplementary Figure 6. Acoustic and optical modes can be identified in the SAF heterostructures with RKKY interaction[^2-4^](#_ENREF_2). The AM and OM mode for out-of-plane situation (easy axis is perpendicular to film plane) could be described by the simplified FMR equations expressed as follows[^2^](#_ENREF_2)^,^[^3^](#_ENREF_3):

$\frac{\omega}{\gamma}=H+H_{k}-4\pi M$ (1)

$\frac{\omega}{\gamma}=H+H_{k}-J_{\mathrm{RKKY}}-4\pi M$ (2)

where *ω* is angular resonance frequency (9.2 Ghz× 2π), *γ* is the gyromagnetic ratio of 2.8 MHz/Oe, *H* is the resonance field, *H*_k_ relates to the volume magnetocrystalline anisotropy, *4πM* is 17.7 kOe for Co at room temperature[^5^](#_ENREF_5). *J*_RKKY_ is the effective RKKY interaction field, which can be determined by the FMR field difference between AM and OM modes directly. A positive J_RKKY_ indicates an AFM coupling whereas a negative *J*_RKKY_ represents a FM coupling[^2^](#_ENREF_2)^,^[^3^](#_ENREF_3). The higher absolute value of *J*_RKKY_ indicates the stronger IEC. According to Eq (1) and (2), resonance field of OM mode is higher than AM mode in AFM coupling (6, 7.5, 9 Å) and becomes opposite while in FM coupling (2, 10 Å) as shown in Supplementary Figure 6(a). Both parallel and antiparallel moment alignments exist in all the FMR spectra, and in most cases, OM mode has weaker FMR field than that of AM mode except *t*_Ru_ = 0.9 Å, indicating a strong antiparallel moment alignment. Supplementary Figure 6(b) and (c) show the IL gating effect for both in-plane and out-of-plane direction, representing a quantitative evidence of voltage control of RKKY interaction. The AM and OM mode for in-plane situation could be described by the following simplified FMR equations[^2^](#_ENREF_2)^,^[^3^](#_ENREF_3):

$\frac{\omega}{\gamma}=\sqrt{(H-H_{k})(H+4\pi M)}$ (3)

$\frac{\omega}{\gamma}=\sqrt{(H-H_{k}-J_{\mathrm{RKKY}})(H-J_{\mathrm{RKKY}}+4\pi M)}$ (4)


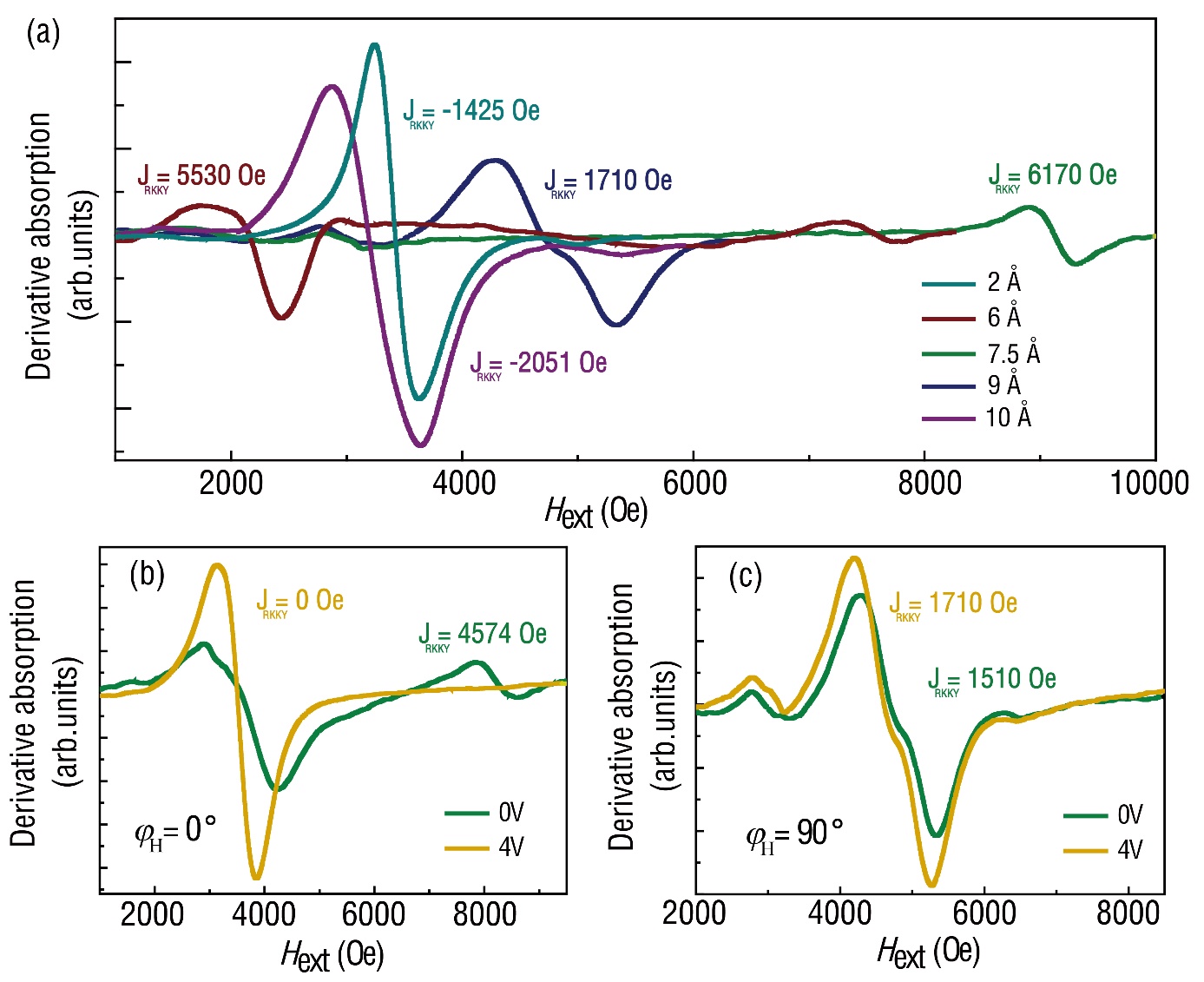


**Supplementary Figure** 8 **|** **In-situ FMR measurement for (Pt/Co)_2_/Ru/(Co/Pt)_2_ SAF structure.** (a) Ru Thickness dependence of FMR spectra in out-of-plane direction at initial state. IL gating effect via IL gating of (b) in-plane direction and (c) out-of-plane direction while *t*_Ru_=0.9 nm. $\varphi_{H}=0^{^{\circ}}$represents in-plane direction while ${90}^{^{\circ}}$ means out-of-plane direction.

**Supplementary Note 8. Domain nucleations vs. H-field while with and without *V*_g_**

Domain regulation with V_g_ was also studied through the nucleation process. In this case, we saturated sample with a large negative H-field, and then increased H-field gradually to locate the positive flip field. The corresponding domain reversals are shown in Supplementary Figure 9(b-g). When H-field changes from the negtive saturation field (-4000 Oe) to positive saturation field (+4000 Oe), the ungated domain nucleations start approximatively at +1700 Oe and finish at +2200 Oe (Supplementary Figure 9(b-d)). After *V*_g_= 4 V is applied, the nucleation field appears just at +600 Oe (Supplementary Figure 9(e)) and the flip process quickly finished at +672 Oe (Supplementary Figure 9(g)). Further increasing H-field to the initial nucleation field (~1900 Oe), no domain nucleations happen at this time as shown in Supplementary Figure 9(h-j). If we take no account of the brightness changes, all the domain images remain the same as saturation state (Supplementary Figure 9(g)). These are far different from that of the initial states shown in Supplementary Figure 9(b-d), indicating that the applied E-field significantly influences the domain evolutions and magnetization reversals.


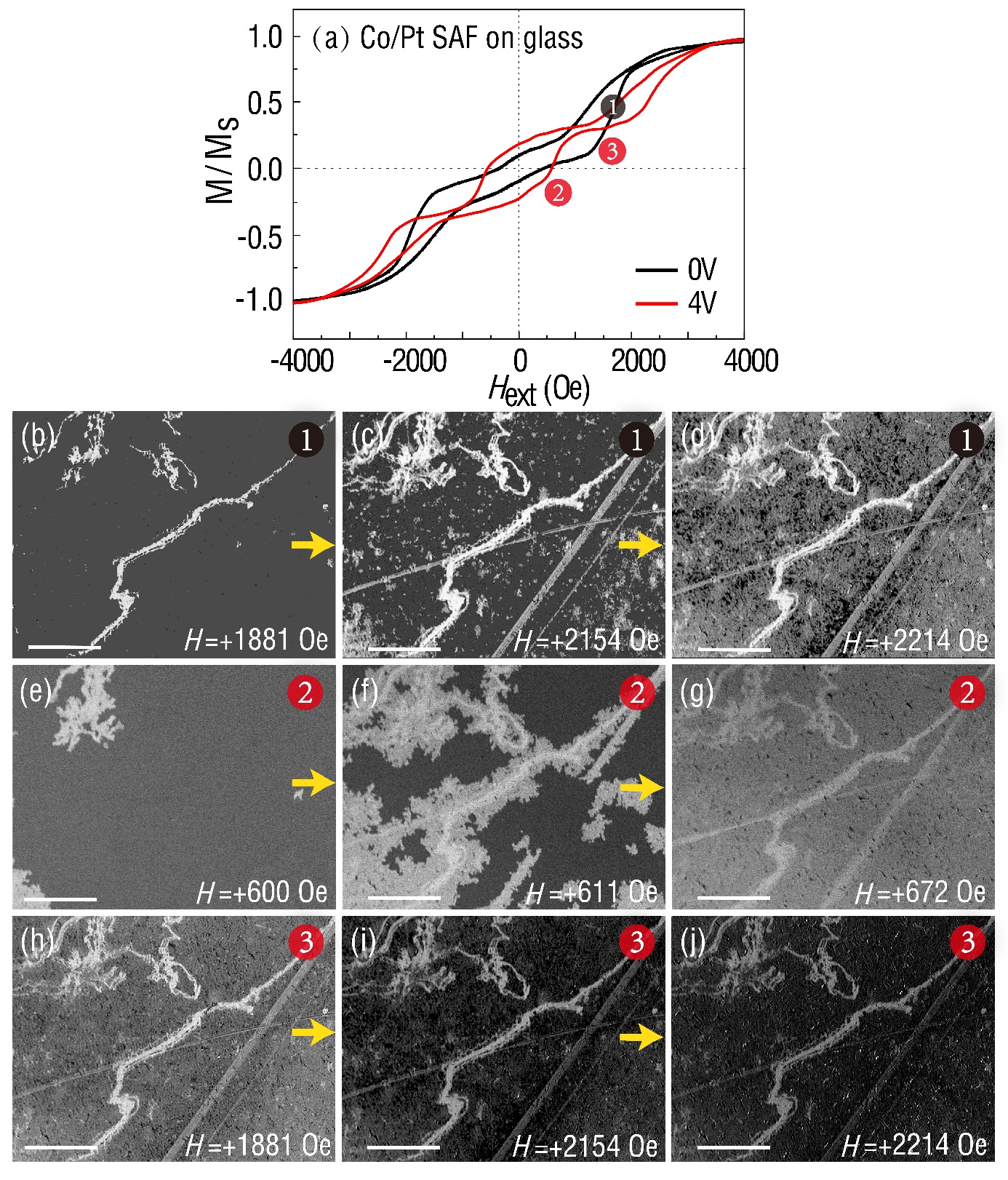


**Supplementary Figure 9 |** **In-situ Kerr microscopy images for IL gating induced** **domain evolutions of (Pt 9 Å/Co 7.5 Å)_2_/Ru (0.91 nm)/(Co 7.5 Å /Pt 9 Å)_2_/glass structure.** (a) Ex situ hysteresis loops tested by VSM. (b-d) Domain nucleations for ungated state. (e-h) Domain evolutions after gating at 4 V. Magnetization reversal finishes at 672 Oe, there are only brightness changes at the original nucleation field +1881 ~2214 Oe. (b-j) Scale bar, 100 μm.

**References**

1 Zhao, S. *et al.* Quantitative Determination on Ionic‐Liquid‐Gating Control of Interfacial Magnetism. *Advanced Materials* **29**, 1606478-n/a (2017).

2 Gong, Y. *et al.* Determination of magnetic anisotropies, interlayer coupling, and magnetization relaxation in FeCoB/Cr/FeCoB. *Journal of Applied Physics* **106**, 063916 (2009).

3 Xing, X. *et al.* RF magnetic properties of FeCoB/Al_2_O_3_/FeCoB structure with varied Al_2_O_3_ thickness. *IEEE Transactions on Magnetics* **47**, 3104-3107 (2011).

4 Li, S. *et al.* Tunable optical mode ferromagnetic resonance in FeCoB/Ru/FeCoB synthetic antiferromagnetic trilayers under uniaxial magnetic anisotropy. *Advanced Functional Materials* **26**, 3738-3744 (2016).

5 Yang, Q. *et al.* Spin-orbital coupling induced four-fold anisotropy distribution during spin reorientation in ultrathin Co/Pt multilayers. *Applied Physics Letters* **110**, 022403 (2017).
