# Supplementary material for: Ionic liquid gating control of RKKY interaction in FeCoB/Ru/FeCoB and (Pt/Co)2/Ru/(Co/Pt)2 multilayers
Source: Nat Commun. 2018 Mar 7;9:991. doi: 10.1038/s41467-018-03356-z (PMC5841336; doi:10.1038/s41467-018-03356-z)
Supplement: Supplementary file 2 — Description of Additional Supplementary Files [file 41467_2018_3356_MOESM2_ESM.pdf]

Description of Additional Supplementary Files

File Name: Supplementary Movie 1: Supplementary dynamic picture for Figure 5
Description: Dynamic picture for Voltage control domain switching
