# Supplementary figures and images for: Ionic liquid gating control of RKKY interaction in FeCoB/Ru/FeCoB and (Pt/Co)2/Ru/(Co/Pt)2 multilayers
Source: Nat Commun. 2018 Mar 7;9:991. doi: 10.1038/s41467-018-03356-z (PMC5841336; doi:10.1038/s41467-018-03356-z)

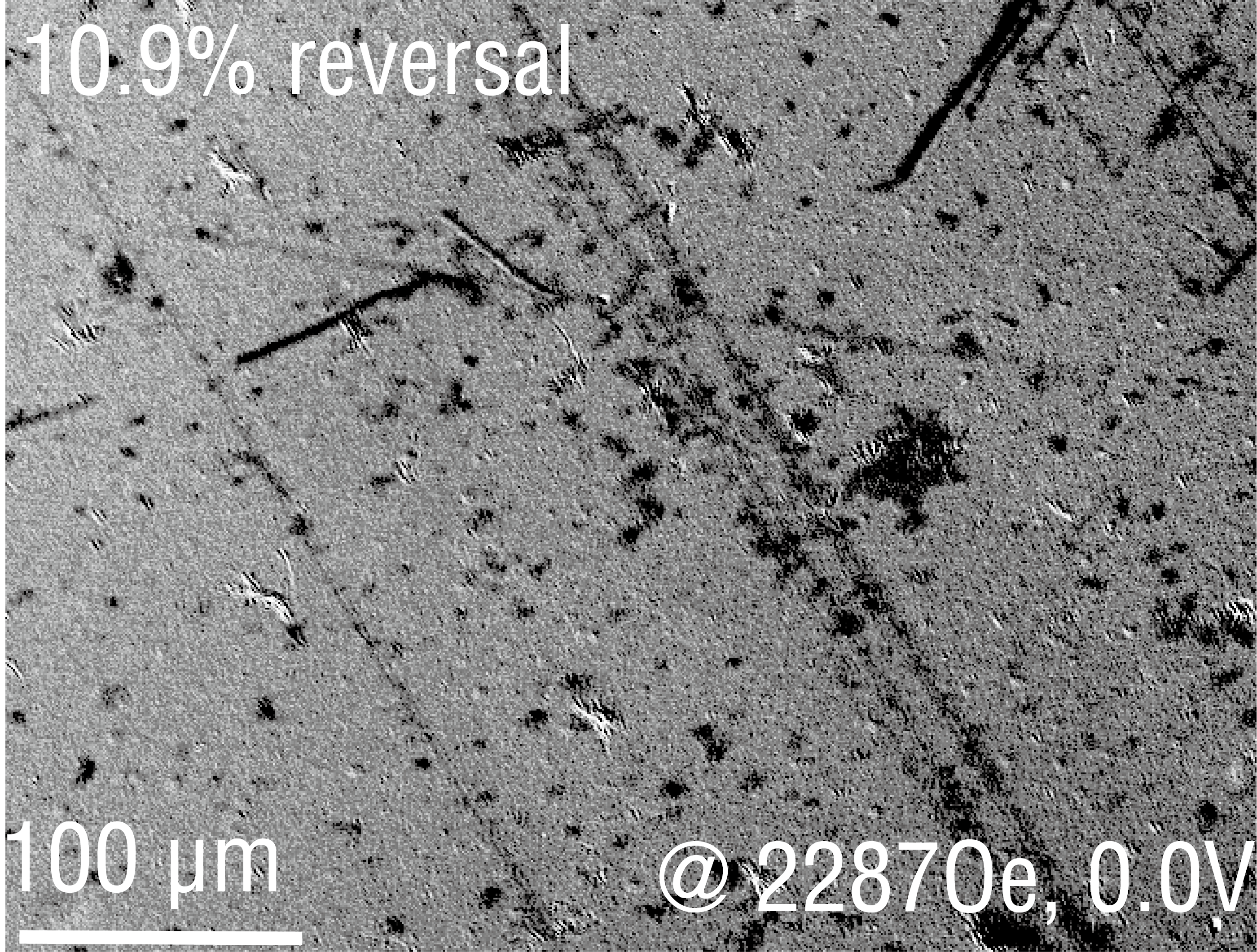

Supplement: Supplementary file 3 — Supplementary Movie 1 [file 41467_2018_3356_MOESM3_ESM.gif]
